# Mapping Research Domain Criteria using a transdiagnostic mini-RDoC assessment in mental disorders: a confirmatory factor analysis

**DOI:** 10.1007/s00406-022-01440-6

**Published:** 2022-07-01

**Authors:** Bernd R. Förstner, Mira Tschorn, Nicolas Reinoso-Schiller, Lea Mascarell Maričić, Erik Röcher, Janos L. Kalman, Sanna Stroth, Annalina V. Mayer, Kristina Schwarz, Anna Kaiser, Andrea Pfennig, André Manook, Marcus Ising, Ingmar Heinig, Andre Pittig, Andreas Heinz, Klaus Mathiak, Thomas G. Schulze, Frank Schneider, Inge Kamp-Becker, Andreas Meyer-Lindenberg, Frank Padberg, Tobias Banaschewski, Michael Bauer, Rainer Rupprecht, Hans-Ulrich Wittchen, Michael A. Rapp

**Affiliations:** 1 Social and Preventive Medicine, Department of Sports and Health Sciences, University of Potsdam, Am Neuen Palais 10, 14469 Potsdam, Germany; 2 Department of Psychiatry and Psychotherapy CCM, Charité, Universitätsmedizin Berlin, Freie Universität Berlin, Humboldt-Universität Zu Berlin, and Berlin Institute of Health, Berlin, Germany; 3 Department of Psychiatry, Psychotherapy and Psychosomatics, Faculty of Medicine, RWTH Aachen University, Aachen, Germany; 4 JARA-Brain, Research Center Jülich, Jülich, Germany; 5 Institute of Psychiatric Phenomics and Genomics (IPPG), University Hospital, LMU Munich, Munich, Germany; 6 University Hospital Düsseldorf, Medical School, Heinrich Heine University Düsseldorf, Düsseldorf, Germany; 7 Department of Child and Adolescent Psychiatry, Psychosomatics and Psychotherapy, Philipps University Marburg, Marburg, Germany; 8 Department of Psychiatry and Psychotherapy, Mannheim, Central Institute of Mental Health, Medical Faculty Mannheim, Mannheim, Germany; 9 Department of Psychiatry and Psychotherapy, University Hospital, LMU Munich, Munich, Germany; 10 Department of Child and Adolescent Psychiatry and Psychotherapy, Central Institute of Mental Health, Medical Faculty Mannheim, Mannheim, Germany; 11 Department of Psychiatry and Psychotherapy, University Hospital Carl Gustav Carus, Medical Faculty, Technische Universität Dresden, Dresden, Germany; 12 Department of Psychiatry and Psychotherapy, University of Regensburg, Regensburg, Germany; 13 Max Planck Institute of Psychiatry, Munich, Germany; 14 Institute of Clinical Psychology and Psychotherapy, Technische Universität Dresden, Dresden, Germany; 15 Translational Psychotherapy, Department of Psychology, Friedrich-Alexander-Universität Erlangen-Nürnberg, Erlangen, Germany; 16 Social Neuroscience Lab, Department of Psychiatry and Psychotherapy, Center of Brain, Behavior, and Metabolism (CBBM), University of Lübeck, Lübeck, Germany

**Keywords:** Diagnosis and classification, Research Domain Criteria, PD-CAN, Confirmatory factor analysis CFA, RDoC, Transdiagnostic

## Abstract

This study aimed to build on the relationship of well-established self-report and behavioral assessments to the latent constructs positive (PVS) and negative valence systems (NVS), cognitive systems (CS), and social processes (SP) of the Research Domain Criteria (RDoC) framework in a large transnosological population which cuts across DSM/ICD-10 disorder criteria categories. One thousand four hundred and thirty one participants (42.1% suffering from anxiety/fear-related, 18.2% from depressive, 7.9% from schizophrenia spectrum, 7.5% from bipolar, 3.4% from autism spectrum, 2.2% from other disorders, 18.4% healthy controls, and 0.2% with no diagnosis specified) recruited in studies within the German research network for mental disorders for the Phenotypic, Diagnostic and Clinical Domain Assessment Network Germany (PD-CAN) were examined with a Mini-RDoC-Assessment including behavioral and self-report measures. The respective data was analyzed with confirmatory factor analysis (CFA) to delineate the underlying latent RDoC-structure. A revised four-factor model reflecting the core domains positive and negative valence systems as well as cognitive systems and social processes showed a good fit across this sample and showed significantly better fit compared to a one factor solution. The connections between the domains PVS, NVS and SP could be substantiated, indicating a universal latent structure spanning across known nosological entities. This study is the first to give an impression on the latent structure and intercorrelations between four core Research Domain Criteria in a transnosological sample. We emphasize the possibility of using already existing and well validated self-report and behavioral measurements to capture aspects of the latent structure informed by the RDoC matrix.

**Supplementary Information:**

The online version contains supplementary material available at 10.1007/s00406-022-01440-6.

## Introduction

Since its launch in 2010, the Research Domain Criteria (RDoC) framework by the National Institute of Mental Health (NIMH) [1] gained traction, in an effort to transgress established symptom based diagnostic systems [Diagnostic and Statistical Manual of Mental Disorders (DSM) [2]/International classification of diseases [ICD] [3]], implementing new categories representing fundamental principles underlying these taxonomies [4]. This transnosological approach aims to understand the full spectrum of mental health and illness through incorporating biological, physiological and behavioral knowledge, while it seeks to overcome existing problems of symptom based heterogeneity, comorbidity, and research limitations induced by diagnostic categories [5–7].

The RDoC approach represents a framework based on behavioral dimensions and neurobiological measures with the final goal of improving classification systems for mental diseases and treatment approaches [1, 8]. This goal arose from a fundamental critique on the DSM-5 and its lack of validity, which is caused by symptom-based diagnosis that do not categorize by etiology and fail to match mechanisms or markers identified in biological psychiatry [9]. These shortcomings have been linked to low response and remission rates in psychopharmacology, furthermore to potential harming of patients with needless treatments and diagnoses [10, 11].

The RDoC matrix [6] offers a systematic overview of currently six core domains forming basic dimensions of human functioning: positive (PVS) [12] and negative valence systems (NVS) [13], cognitive systems (CS) [14], social processes (SP) [15], arousal and regulatory systems (ARS) [16] and sensorimotor systems (SMS) [17]. For each domain a hierarchical system of constructs and subconstructs is defined to cover specific facets of this domain. Each (sub-)construct has eight “units of analysis” representing methodological aspects to integrate the following levels of information covering genes, molecules, cells, circuits, physiology, behavior, self-report, and paradigms. In this current study, only four domains are being investigated since ARS and SMS were subsequently added to the matrix after data collection started. The domains, (sub-)constructs and units of analysis investigated in the current work, are described in detail in the methods section.

Little work has been done to validate these RDoC defined core domains using the units self-report and behavioral investigation, while the existing literature on this subject shows large variations of methodological approaches and definitions on these functional core domains. Recent studies using self-report measures explored or confirmed the multi-factorial structure of the PVS domain and showed connection to common constructs of personality [18, 19]. In addition, a specific PVS-scale and a subscale for empathy were implemented and validated [18, 20]. In a purely psychometric approach, Tsanas et al. (2017) found valid subconstructs assessing the latent structure of mood symptoms that further validate the RDoC construct PVS [21]. Paulus et al. [22] used an RDoC framework with self-report and behavioral measures to define the domains NVS and PVS and provided evidence suggesting that both domains should be treated independently and not as two sides of the same coin [22]. Their findings supported the assumption of an independent reward neural circuit [23].

Regarding the NVS domain, a review of self-report measures concluded that more exploratory work needs to be conducted to develop valid instruments to measure this domain and its subconstructs [24]. Nonetheless, a confirmatory study in children with internalizing disorder symptoms using self-report measures revealed an “higher order NVS” with a multifactorial internal structure, supporting the idea of a latent NVS domain in developing children and suggesting an underlying set of biological mechanisms with construct specific elements [25].

Until this date, no validation studies regarding the CS domain exist, that use an explicit RDoC framework for self-report or behavioral measures. However, the sub-constructs integrating the domain have been investigated fairly well and have strong neurobiological support [26]. Furthermore, a latent cognitive multifactorial structure common in subjects diagnosed with schizophrenia, bipolar disorder, and healthy adults has been validated which supports the idea of a cognitive multifactorial system congruent to the propositions of the RDoC framework [27]. Recent research on the SP domain showed promising results on capturing dimensional SP constructs with an already existing self-report measure in children with normative development and autism spectrum disorder [28].

In summary, single domains forming the latent structure of the RDoC framework regarding self-report measures have partially been substantiated. Following this research and the current recommendations by the NIMH [29, 30] our study sought to investigate the RDoC framework spanning across four of the core domains for the first time.

Specifically, the goal of the current study was to establish a first look at the latent constructs of PVS, NVS, CS, and SP and their relationship using already existing self-report and behavioral assessments in a transnosological mixed population which cuts across DSM-V/ICD-10 disorder criteria categories. Moreover, we aimed to improve our understanding about the characteristics of these latent variables and their intercorrelations.

## Methods

### Participants and procedures

Overall, 1912 participants were recruited for the Phenotypic, Diagnostic and Clinical Domain Assessment Network Germany (PD-CAN) within the German research network for mental disorders [31]. All patients were initially recruited for specific intervention and observation studies of a given disease entity within each of the nine network consortia. Specifically, in this study, we report data from eight research consortia (PROTECT-AD, ESCAlife, ASD-Net, BipoLife, OptiMD, GCBS, APIC, ESPRIT). Since the main cohort of the AERIAL project focusing on the development of substance use disorders represents a primarily adolescent at risk sample with comparably low prevalence of mental disorders, measuring similar domains using partially different assessment methods, we excluded data from this consortium from the present analyses given the focus on confirmatory factor analysis. Due to the heterogeneity of the studies within the network, each of the projects implemented specific in- and exclusion criteria as well as the Mini-RDoC assessment in toto or partially depending on the individual assessment fit in the respective study (see SI1 or below for details). An overview on the main aims, sampling including in-/exclusion criteria and number of participants included for each specific study is available in supplementary material SI2. All subjects gave additional written informed consent to participate in and contribute their data to the PD-CAN network in an anonymized fashion at the local sites. Data were transmitted from the partner site via secure servers and data carriers, or an anonymized electronic research file implemented in secuTrial^®^ (interActive Systems GmbH, Berlin).

A consented test-battery with 16 psychological tests was administered after recruitment in addition or embedded into the usual testing of each study, with the principal aim to measure behavioral and self-report constructs of the RDoC matrix [1, 4, 6]. The battery comprises a shell model (Fig. SI1) with two layers and a core. Baseline implementation of the core variables was obligatory and shell variables were optional depending on their fit to the specific assessment process (f.e. questionnaire processing time) to accommodate the individual study designs. The consent process on the battery was managed through a Delphi process including experts from each of the nine consortia and resulted in a selection of tasks for shell and core assessments in 2014. The consent group also determined and assigned a priori the domains assessed within the RDoC matrix by using the information given by the NIMH and publications on self-report/behavioral measures within the RDOC framework at that time [12–16, 22, 25, 32]. Supplementary Table SI3 shows a detailed description of this battery and the tests. The derived 29 variables/scales included in the model were implemented to assess PVS, NVS, CS und SP as latent factors.

### Measurements

Positive and negative valence systems: the Positive and Negative Affect Schedule (PANAS) [33] is a 10-item self-report scale on positive and negative affect. The Behavioral Inhibition and Activation Scale (BIS/BAS) [34] includes 24-items assessing motivation towards goal-motivated or avoidance of aversive outcomes. The BSI-53 (Brief Symptom Inventory) [35] is a self-report psychometric instrument to assess a broad range of psychological problems and symptoms of psychopathology. It consists of 53 items yielding 9 scores for primary symptom dimensions and three global distress indices. Abuse and neglect during childhood and adolescence was measured using the 5-item Childhood Trauma Screener (CTS) [36].

Cognitive systems: the two-tiered TMT A/B [37] is a widely used neuropsychological instrument that measures speed of scanning, visuomotor tracking, divided attention and cognitive flexibility. Also included were two subtests of the Wechsler adult intelligence scale (WAIS-IV) [38]. First, Digit-span Forward task (DF) assesses working memory capacity by asking participants to recall an increasing sequence of spoken digits. Second, the Digit Symbol Substitution Test (DSST) measures cognitive processing speed, short term memory, learning ability, visual perception, visuomotor coordination, ability for visual scanning and attention. The short form of the Barratt Impulsiveness Scale (BIS-15) [39] consists of 15 items assessing the behavioral or personality construct of impulsiveness represented in three subscales of non-planning, attentional and motor impulsivity. Lastly, the Multiple-choice Word Test (MWT-B) [40] with 37 items offers an estimate for premorbid cognitive ability.

Social processes: the WHO disability assessment schedule 2.0 (WHO-DAS 2.0) [41] is a 12-item instrument developed by the WHO for assessing health status and disability. Specifically, the single item reflecting social integration was used. Similarly, additional subscales from the BSI-53 [35] assessing social relationships and social anhedonia were used. The emotion regulation questionnaire (ERQ) [42] is a 10-item scale assessing two emotion regulation strategies, cognitive reappraisal and expressive suppression, in relation to SP. In addition, three sociodemographic variables were implemented to include indirect measures of existing social relations, affiliation and attachment. Used subscales and their relation to the RDoC matrix are shown in Table 1.

**Table 1:** A priori allocation of the PD-CAN assessment to RDoC

Measured RDoC and constructs	Instrument	
**Positive valence systems (PVS)**		
Reward responsiveness	PANAS	Subscale positive affect
Reward responsiveness	BIS/BAS	Subscale reward responsiveness
Reward valuation	BIS/BAS	Subscale drive
Reward learning	BSI-53	Single items obsessive–compulsive
**Negative valence systems (NVS)**		
Potential threat (anxiety)	BIS/BAS	Subscale behavioral inhibition
Potential threat (anxiety)	BSI-53	Subscale anxiety
Potential threat (anxiety)	BSI-53	Subscale phobic anxiety
Potential threat (anxiety)	BSI-53	Subscale somatization
Frustrative nonreward	BSI-53	Subscale hostility
Loss	BSI-53	Single item anhedonia
Sustained threat	CTS	Sumscore childhood trauma
**Cognitive systems (CS)**		
Language	MWT-B	Raw score multiple-choice word test—version B
Attention	TMT-A	Raw score trail making test—version A
Cognitive control	TMT-B	Raw score trail making test—version B
Cognitive control	BIS-15	Subscale attentional impulsivity
Cognitive control	BIS-15	Subscale non-planning impulsivity
Working memory	DF	Sumscore digit span forward
Working memory	DSST	Raw score digit symbol substitution test
**Social processes (SP)**		
Affiliation and attachment	Demography	Single item graduation
Affiliation and attachment	Demography	Single item occupation
Affiliation and attachment	Demography	Single item residential status
Affiliation and attachment	WHO-DAS 2.0	Single item Friendships reversed
Affiliation and attachment	BSI-53	Subscale interpersonal sensitivity
Affiliation and attachment	BSI-53	Single item social anhedonia
Perception and understanding of self	BSI-53	Subscale paranoid ideation
Perception and understanding of self	ERQ	Subscale reappraisal
Perception and understanding of self	ERQ	Subscale suppression

*BIS-15* Barratt Impulsiveness Scale—Short Form, *BIS/BAS* Behavioral Inhibition System/Behavioral Activation System Scale, *BSI-53* Brief Symptom Checklist, *CTS* Childhood Trauma Screener, *DF* Digit span forward, *DSST* Digit Symbol Substitution Test, *ERQ* Emotion Regulation Questionnaire, *PANAS* Positive and Negative Affect Scale, *WHO-DAS 2.0* WHO Disability Assessment Schedule 2.0

The structure of data showed heterogeneous missingness (36.68% throughout the whole raw dataset). To deal with missingness, we applied the following strategy: first, we excluded all participants (*N* = 481) lacking all the indicator variables/scales for at least one of the four RDoC from our analyses. Overall missingness was thus reduced by 12.69%. Missing values within the observed variables (see Table 2) that were considered (a priori) for the factor analysis by the consent group amounted to 11.12%. Even though the overall missing rate can be considered as typical [43], in a second step, we considered individual items exhibiting missingness in more than 35% of data. Four specific variables retained missingness at approximately 39%: BIS/BAS subscales Behavioral Inhibition, BAS-Drive, BAS-Reward Responsiveness and PANAS Positive Affect (see also Table 2). Given that three of the indicators had been selected for PVS, we decided to use three of the six single items from the BSI-53 Obsessive–compulsive scale (focusing on inhibition and habituation as parts of PVS [12]) instead of the whole scale as indicators in order to strengthen the database informing the latent factor PVS. This benefited the full information maximum likelihood method (FIML) [44] used to handle missing data, because more detailed information was available for missing variable estimation. The final sample to evaluate the structure of our four-factor model consisted of *n* = 1431 participants. A descriptive overview on the demographic and diagnostic information of the final sample can be found in Table 3.

**Table 2:** Descriptive Statistics of Observed Variables (untransformed)

Variable	Instrument	Mean	SD	Min	Max	Mdn	IQR	% Missing
Subscale positive affect (PVS)	PANAS	16.39	7.56	0.00	42.00	16.00	10.00	38.50
Subscale reward responsiveness (PVS)	BIS/BAS	10.27	2.53	4.00	20.00	10.00	3.00	39.20
Subscale drive (PVS)	BIS/BAS	8.94	2.42	4.00	16.00	9.00	4.00	39.20
Single item (15) obsessive–compulsive (PVS)	BSI-53	1.38	1.20	0.00	4.00	1.00	2.00	0.63
Single item (26) obsessive–compulsive (PVS)	BSI-53	0.48	0.88	0.00	4.00	0.00	1.00	0.35
Single item (27) obsessive–compulsive (PVS)	BSI-53	1.16	1.13	0.00	4.00	1.00	2.00	0.28
Subscale behavioral inhibition (NVS)	BIS/BAS	12.02	3.63	7.00	26.00	12.00	6.00	39.20
Subscale anxiety (NVS)	BSI-53	0.87	0.76	0.00	3.83	0.67	1.13	0.35
Subscale phobic anxiety (NVS)	BSI-53	0.73	0.85	0.00	4.00	0.40	1.20	0.35
Subscale somatization (NVS)	BSI-53	0.60	0.62	0.00	3.57	0.43	0.71	0.35
Subscale hostility (NVS)	BSI-53	0.56	0.60	0.00	3.40	0.40	0.60	0.35
Single item anhedonia (NVS)	BSI-53	0.93	1.17	0.00	4.00	1.00	1.00	0.42
Sumscore childhood trauma (NVS)	CTS	2.91	3.30	0.00	19.00	2.00	4.00	1.05
Raw score MWT-B (CS)	MWT-B	27.85	5.17	0.00	37.00	28.00	6.00	11.11
Raw score TMT-A (CS)	TMT-A	28.17	11.96	10.00	114.00	25.19	12.61	18.24
Raw score TMT-B (CS)	TMT-B	61.88	27.99	15.33	282.00	55.00	26.24	16.00
Subscale attentional impulsivity (CS)	BIS-15	5.34	2.88	0.00	14.00	5.00	4.00	12.86
Subscale non-planning impulsivity (CS)	BIS-15	6.32	3.13	0.00	15.00	6.00	4.00	12.86
Sumscore DF (CS)	DF	8.33	2.35	2.00	16.00	8.00	3.00	17.12
Raw score DSST (CS)	DSST	− 0.33	1.07	− 4.91	4.26	− 0.34	1.35	8.18
Single item graduation (SP)		5.67	1.67	0.00	8.00	7.00	3.00	0.49
Single item occupation (SP)		0.64	0.48	0.00	1.00	1.00	1.00	2.94
Single item residential status (SP)		0.61	0.49	0.00	1.00	–	–	0.42
Single item friendships (*r*) (SP)	WHO-DAS 2.0	2.90	1.16	0.00	4.00	3.00	2.00	29.63
Subscale interpersonal sensitivity (SP)	BSI-53	0.98	0.97	0.00	4.00	0.75	1.25	0.28
Single item social anhedonia (SP)	BSI-53	0.90	1.13	0.00	4.00	1.00	1.00	0.28
Subscale paranoid ideation (SP)	BSI-53	0.67	0.74	0.00	4.00	0.40	1.00	0.42
Subscale reappraisal (SP)	ERQ	24.34	7.36	3.00	42.00	25.00	9.00	15.72
Subscale suppression (SP)	ERQ	15.56	5.07	2.00	28.00	16.00	7.00	15.72

*SD* Standard deviation, *Mdn* Median, *IQR* Interquartile range, *r* reversed, *PVS* Positive valence systems, *NVS* Negative valence systems, *CS* Cognitive systems, *SP* Social processes, *BIS-15* Barratt Impulsiveness Scale—Short Form, *BIS/BAS* Behavioral Inhibition System/Behavioral Activation System Scale, *BSI-53* Brief Symptom Checklist, *CTS* Childhood Trauma Screener, *DF* Digit span forward, *DSST* Digit Symbol Substitution Test, *ERQ* Emotion Regulation Questionnaire, *PANAS* Positive and Negative Affect Scale, *TMT-A/B* Trail Making Test A/B, *WHO-DAS 2.0* WHO Disability Assessment Schedule 2.0, all variables were plausibility checked: scores were in range of respective assessment

**Table 3:** Total sample characteristics and consortia sample details

Variable	Total sample	PROTECT-AD	ESCAlife	ASD-NET	BipoLife	Opti-MD	GCBS	APIC	ESPRIT
Subjects, used in analysis, *n*	1431	600	25	23	99	171	40	89	384
Demographic characteristics									
Age, years									
MD ± SD	34.6 ± 12.5	33.0 ± 11.2	27.6 ± 7.5	26.4 ± 5.1	35.4 ± 12.5	43.2 ± 15.3	37.4 ± 14.5	35.7 ± 11.8	33.8 ± 11.7
Range	15–78	15–68	18–43	19–38	18–69	18–78	20–64	18–61	18–65
Missing data, %	0.3	–	–	–	–	1.2	–	–	–
Gender, %									
Female	50.2	55.3	24.0	–	54.5	51.4	47.5	39.3	49.0
Male	49.8	44.7	76.0	100.0	45.5	48.3	52.5	60.7	51.0
Marital status, %									
Single	34.6	38.8	52.0	78.3	66.7	46.2	67.5	66.3	–
Married/partnership	30.7	53.0	36.0	21.7	18.2	35.7	22.5	22.5	–
Separated	4.3	1.3	8.0	–	6.1	7.6	–	2.2	–
Divorced	5.2	6.5	4.0	–	8.1	9.9	7.5	7.9	–
Widowed	0.3	0.3	–	–	–	0.6	2.5	–	–
Missing data	27.0	–	–	–	1.0	–	–	1.1	100.0
Migration, %									
Yes	26.1	25.8	32.0	8.7	33.3	15.2	45.0	44.9	24.2
No	70.9	73.8	68.0	91.3	66.7	33.9	55.0	53.9	75.0
Missing data	3.0	0.3	–	–	–	22.8	–	1.2	0.8
Graduation^a^, years									
MD ± SD	11.7 ± 1.5	11.6 ± 1.5	11.1 ± 1.5	12.4 ± 1.0	12.1 ± 1.4	11.8 ± 1.6	11.8 ± 1.7	11.2 ± 1.7	12.0 ± 1.4
Missing data, %	9.1	2.3	8.0	–	2.0	8.2	5.0	6.7	1.3
Occupation, %									
Unemployed	35.0	30.8	32.0	43.5	53.5	33.9	50.0	53.9	31.0
Employed	62.1	69.2	68.0	56.5	46.5	43.3	50.0	44.9	68.5
Missing data	2.9	–	–	–	–	22.8	–	1.1	0.5
Net income, €, %									
Up to 500	7.5	–	20.0	34.8	29.3	15.2	17.5	37.1	–
500–1000	7.3	–	36.0	43.5	20.2	16.4	27.5	31.3	–
1000–2000	8.4	–	28.0	8.7	33.3	26.3	30.0	23.6	–
2000–3000	2.2	–	12.0	–	9.1	7.0	15.0	2.2	–
3000–4000	1.0	–	–	–	3.0	5.3	5.0	2.2	–
Over 4000	1.1	–	4.0	4.3	4.0	1.2	2.5	–	–
Missing data	72.3	100.0	–	8.7	1.0	25.1	2.5	4.5	100.0
Municipality size classes, *n*, %									
Up to 5000	8.2	11.3	44.0	4.3	6.1	18.1	0.9	–	–
Up to 20,000	7.0	9.2	8.0	8.7	16.2	12.3	3.5	–	–
Up to 50,000	5.0	6.3	12.0	4.3	11.1	7.6	5.2	–	–
Up to 100,000	8.2	17.3	4.0	4.3	6.1	2.9	27.0	–	–
Up to 500,000	11.9	16.3	32.0	60.9	21.2	17.0	–	–	–
Over 500,000	23.5	38.2	–	17.4	39.4	20.5	63.5	–	–
Missing data	36.2	1.3	–	–	–	21.5	–	100.0	100.0
Clinical characteristics									
Primary diagnosis, %									
AD	42.1	100.0	–	–	2.0	–	–	–	–
MDD	18.1	–	4.0	–	25.3	83.0	100.0	9.0	11.2
SZ	7.9	–	–	–	–	0.6	–	71.9	12.5
BD	7.8	–	–	–	60.6	5.8	–	–	10.7
ASD	3.4	–	–	52.2	–	–	–	–	9.6
PD	0.1	–	–	–	2.0	–	–	–	–
ADHD	1.7	–	96.0	–	–	–	–	–	–
SUD	0.3	–	–	–	4.0	–	–	–	–
PTSD/AjD	0.1	–	–	–	–	0.6	–	–	–
HC	18.4	–	–	47.8	5.1	9.4	–	19.1	55.7
Missing data	0.2	–	–	–	1.0	0.6	–	–	0.3
Comorbidity, %									
Yes	45.2	87.8	36.0	4.3	15.2	17.0	5.0	1.1	16.4
No or not collected	54.8	12.2	64.0	95.7	84.8	83.0	95.0	98.9	83.6
Psychotropic drugs, %									
Yes	51.2	49.0	–	–	76.8	85.4	100.0	57.3	32.6
No	45.4	51.0	100.0	100.0	22.2	4.1	–	9.0	67.4
Missing data	3.4	–	–	–	1.0	10.5	–	33.7	–

*M* Mean, *SD* Standard deviation. Disorder: *AD* Anxiety disorders, *ADHD* Attention deficit hyperactivity disorders, *AjD* Adjustment disorders, *ASD* Autism spectrum disorders, *BD* Bipolar spectrum disorders (Type I/II), *MDD* (Major) Depressive disorders, *PD* Personality disorders, *PDD* Persistent depressive disorders (dysthymia, cyclothymia), *PTSD* Posttraumatic stress disorders, *SUD* Substance use disorders, *SZ* Schizophrenia spectrum disorders

^a^Graduation: 9 years Certificate of Secondary Education [Hauptschulabschluss], 10 years General Certificate of Secondary Education [Realschulabschluss] or Polytechnic degree [Abschluss der allgemeinbildenden Polytechnischen Oberschule der ehemaligen DDR], 12 years Technical-diploma [Fachabitur, Fachhochschulreife, Fachgebundene Hochschulreife], 13 years University-entrance diploma [Abitur, Allgemeine Hochschulreife], missing data still in school, other types of school (e.g., school for handicapped children), school dropout or missing data

### Statistics

The underlying latent RDoC factor structure of the PD-CAN assessment was tested using confirmatory factor analysis (CFA) with Maximum likelihood (ML) estimation. Specifically, we fit the confirmatory four-factor model using lavaan version 0.6–6 in R version 4.0.2 with RStudio 1.3.1073 with FIML [44] handling missing data which was considered missing at random (MAR). Latent factors were standardized, i.e., variance was restricted to 1, allowing free estimation of all factor loadings. In addition, the four-factor model was compared to a one-factor solution using the same variables, which sets the correlation between the latent factors to 1 and another model which doesn’t allow covariances between the latent factors treating them as independent.

Given that exploratory data analysis with the Shapiro–Wilk Test [45] for multivariate normal distribution revealed that none of the indicator variables were normally distributed. We used natural logarithm (ln) for right skewed variables or Johnson transformation [46] for variables with a high range to adjust the distribution. Variables/scales that were already z-transformed (DSST score) or were dichotomous or categorical (e.g., residential status) did not undergo transformation. To ensure a congruent polarity for SP we reversed the WHO-DAS 2.0 single item ‘friendships’ since all utilized indicators for SP had negative polarity. Raw scores for all observed variables as well as item-specific missingness are provided in Table 2.

## Results

Confirming the primary hypothesis, all indicators showed significant (*p* < 0.001, except BIS-15 Non-planning Impulsivity scale with *p* < 0.05) factor loadings on the considered domains with standardized coefficients ranging from − 0.76 to 0.49 for PVS, − 0.53 to 0.85 for NVS, − 0.79 to 0.71 for CS and − 0.92 to 0.54 for SP. However, the four-factor model fit (model 1) was poor with a comparative fit index (CFI) of 0.77, a Tucker–Lewis index (TLI) of 0.75 and a root mean square error of approximation (RMSEA) of 0.078 with a 90% confidence interval (CI) (0.076–0.081). However, compared to a single factor solution (model 2: *χ*^2^(6) 1158.1, < 0.001) or a solution assuming the four factors as independent (model 3: *χ*^2^(6) 2571.2, *p* < 0.001), fit was significantly better. For more information on all analyzed CFA-models see supplementary Table SI5 for details.

To address poor fit, we examined the amount of variance explained by each variable/scale using *R*-Squared estimates for each indicator. Twelve indicators explained less than 20% of the variance in the respective domain and were excluded from further analyses (see Table 4).

**Table 4:** Dropped indicators with low R-square

RDoC	Instrument	Variable	Estimate
PVS	BIS/BAS	Subscale reward responsiveness	.074
PVS	BIS/BAS	Subscale drive	.121
NVS	CTS	Sumscore childhood trauma	.088
CS	MWT-B	Raw score MWT-B	.119
CS	BIS-15	Subscale attentional impulsivity	.005
CS	BIS-15	Subscale non-planning impulsivity	.055
CS	DF	Sumscore DF	.114
SP	Sociodemographic	Single item graduation	.013
SP	Sociodemographic	Single item occupation	.022
SP	Sociodemographic	Single item residential status	.009
SP	ERQ	Subscale reappraisal	.083
SP	ERQ	Subscale suppression	.041

Cut-off (*r*^2^ < .20)

*RDoC* Research Domain Criteria, *PVS* Positive valence systems, *NVS* Negative valence systems, *CS* Cognitive systems, *SP* Social processes, *BIS-15* Barratt impulsiveness scale—short form, *BIS/BAS* Behavioral inhibition system/behavioral activation system scale, *CTS* Childhood trauma screener, *DF* Digit span forward, *ERQ* Emotion regulation questionnaire, *MWT-B* Multiple-choice word test-version B

In addition, using modification indices (mi) as a starting point, we reconfigured the model for two indicators. Modification indices reflect a test for covariance across the four factors (RDoC) under study in CFA when covariances are fixed a priori. Specifically, we defined that all variables with a mi > 200, that showed larger covariance of an indicator to another factorial domain were changed to the other domain. In a first step, we relocated the BSI53 Anhedonia item (mi = 270.30) to PVS (instead of NVS). This change was informed by research that suggests anhedonia to be strongly associated with general approach behavior [19], a decrease in positive affect [47] and the reward system as a core component of PVS [48, 49]. In a second step, the BSI53 Hostility subscale (mi = 203.40) was moved to indicate SP since examining the scale items revealed proximity to interpersonal hostility and significant distance to frustrative nonreward to which it was assigned a priori. Subsequently, all modification indices ranged below 200.

Finally, we re-examined the scales on item level that were removed for low *R*-squares earlier to test for an increased shared variance in the latent structure altered based on modification indices. To maximize explained variance, we formed mean scores based on these items. Shared variance significantly improved when we combined items from the BIS/BAS Drive and Reward Responsiveness subscales. Additionally, we also restricted items in the PANAS Positive Affect subscale to reflect hedonic items, items from the BSI Obsessive–Compulsive subscale to items reflecting habituation, and items from the BIS Behavioral Inhibition subscale to reflect anxiety and threat more closely. All changes can be found in Table SI4.

Applying these changes created a significantly improved model fit CFI of 0.93, TLI of 0.92. and RMSEA of 0.077 with 90% CI (0.072–0.082). The overall fit as estimated with the CFI now indicated good fit [50]. This full four factor model (model 4) again fitted the data significantly better than a single factor solution (model 5: *χ*^2^(6) = 1656.3, *p* < 0.001) or the solution with four independent factors (model 6: *χ*^2^(6) = 2327.8, *p* < 0.001).

Regarding the relationship between the indicators and their latent factors, highly significant factor loadings suggest that participants with higher scores in PVS tended to have more hedonic affect (*β* = 0.545, *p* < 0.001), better habituation (*β* = 0.810 *p* < 0.001) and less anhedonia (*β* =  − 0.758, *p* < 0.001).

Participants with high scores in NVS tended to have higher levels of general (*β* = 0.909, *p* < 0.001) and phobic anxiety (*β* = 0.812, *p* < 0.001), more somatization (*β* = 0.746, *p* < 0.001), and more anxiety-based behavioral inhibition (*β* = 0.491, *p* < 0.001).

As expected, participants with higher cognitive abilities exhibited better premorbid intelligence (*β* = 0.688, *p* < 0.001), better cognitive speed processing (*β* =  − 0.796, *p* < 0.001) and executive functioning (*β* =  − 0.796, *p* < 0.001).

Higher scores in the SP domain aligned with better skills in keeping friendships (*β* = 0.532, *p* < 0.001) and less paranoid ideation (*β* =  − 0.787, *p* < 0.001), less social anhedonia (*β* =  − 0.756, *p* < 0.001), as well as less interpersonal sensitivity (*β* =  − 0.909, *p* < 0.001) and hostility (*β* =  − 0.764, *p* < 0.001).

**Fig. 1:**
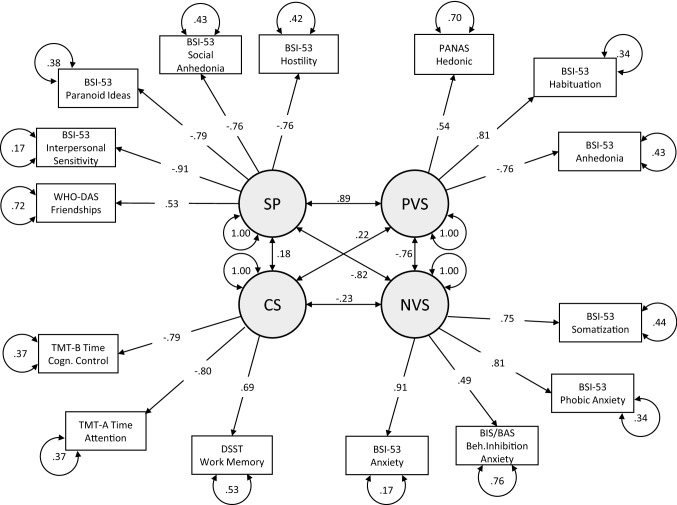
Factorial loadings of the final model on the four Research Domain Criteria. Standardized latent variables: *PVS* Positive valence systems, *NVS* Negative valence systems, *CS* Cognitive systems, *SP* Social processes, Manifest variables: *BIS/BAS Behavioral Inhibition Anxiety* Anxiety based inhibition aspects of BIS/BAS subscale Behavioral Inhibition, *BSI-53 Anhedonia* BSI-53 single item Anhedonia, *BIS-53 Anxiety* BIS-53 subscale Anxiety, *BSI-53 Habituation* habituational aspects of BSI-53 single items Obsessive–compulsive, *BSI-53 Hostility* BSI-53 subscale Hostility, *BSI-53 Interpersonal Sensitivity* BSI-53 subscale Interpersonal Sensitivity, *BSI-53 Phobic Anxiety* BSI-53 subscale Phobic Anxiety, *BSI-53 Paranoid Ideas* BSI-53 subscale Paranoid Ideation, *BSI-53 Social Anhedonia* BSI-53 single Item Social Anhedonia, *BSI-53 Somatization* BSI-53 subscale Somatization, *DSST Work Memory* Digit Symbol Substitution Test raw score, *PANAS Hedonic* Hedonic aspects of PANAS subscale Positive Affect, *TMT-A Time Attention* Trail Making Test—Version A completion time, *TMT-B Time Cognitive Control* Trail Making Test—Version B completion time, *WHO-DAS Friendships* WHO-DAS 2.0 Single item Friendships reversed

There were also significant relations between the four latent factors in that participants with positive affectivity had higher social (*β* = 0.891, *p* < 0.001) and cognitive skills (*β* = 0.221, *p* < 0.001) and less negative affect or aversion against events, objects, and situations (*β* =  − 0.757, *p* < 0.001). At the same time, participants with high levels of negative affect exhibited decreased cognitive (*β* =  − 0.232, *p* < 0.001) and social skills (*β* =  − 0.818, *p* < 0.001). Finally, higher cognitive skills were related to better social skills (*β* = 0.175, *p* < 0.001, see Fig. 1 for details).

## Discussion

The present study used CFA to delineate four core domains of the RDoC framework using behavioral and self-report assessments in a heterogeneous sample of patients suffering from mental disorders and controls. Following the implementation of a short and efficient Mini-RDoC-Assessment approach for this task in multiple studies from within the German Research Network for mental disorders, it was expected to identify latent constructs shared by multiple disorders that may eventually generate a better understanding of the transnosological structure formed by the RDoC framework.

The four-factor model reflecting the core domains PVS and NVS as well as CS and SP showed good fit across a sample of clinical and nonclinical participants spanning across major mental disorder diagnoses supporting the potential transnosological validity of the RDoC framework as implemented using behavioral assessments only. Compared to a one factor solution and a version treating all factors as independent, it also showed significantly better fit.

Specifically, regarding PVS, hedonic (PANAS) and anhedonic aspects of reward responsiveness as well as habituation (BSI-53) connected with reward learning as part of this domain could be confirmed. However, items reflecting reward valuation and reward responsiveness had to be excluded because of high levels of error variance indicating poor fit with the overall construct of PVS. At the same time, anhedonia showed to be a valid indicator of the dimension forming PVS as compared to NVS. These results correspond to previous findings for this construct [24].

For NVS, especially potential threat indicators (BSI-53, BIS/BAS) remained valid within the overall factorial structure. Interestingly, hostility shared more variance with SP than with NVS as a measure for frustrative nonreward.

For CS, behavioral measurements for attention (TMT-A), cognitive control (TMT-B) and working memory (DSST) confirm these constructs as informative for this latent factor. Presumably because of measurement invariance, the self-report measures for subconstructs of cognitive control failed to contribute to the model.

As for SP, this domain could be best represented by the a priori set variables. Despite the observation that almost half of the measures had high levels of error variance (sociodemographic and emotion regulation) and in result had to be removed as indicators from the model (see [22] for similar results), the final model represents a clear representation of SP including social anhedonia (BSI-53), the ability to maintain friendships (WHO-DAS), as well as interpersonal sensitivity and paranoid ideation (BSI-53).

Across domains, a strong connection between the domains PVS, NVS and SP could be substantiated, indicating a universal latent structure spanning across known nosological entities. CS showed smaller but meaningful correlations with the other domains, suggesting that the associations of cognitive abilities with key aspects of affectivity and SP are small and may be moderated by specific disease mechanisms in e.g. schizophrenia [51], autism spectrum disorders [52, 53] and affective disorders [54].

Concerning all removed subconstructs and their measurements, further research needs to be conducted examining existing self-report measures and their allocation inside the RDoC framework as well as to conceptualize new comprehensive measurement tools improving valid measurement of its dimensional latent structure for better adoption in clinical assessment and research.

Finally, our findings suggest that the Mini-RDoC test battery, specifically subsets of the self-report questionnaires BSI-53, PANAS, BIS/BAS, WHO-DAS and TMT A/B and DSST as cognitive tests, successfully resemble aspects of the four core domains measured. Revealing a latent factor structure common to all mental disorders included in this study, as anticipated by the RDoC framework, and gives space for a definition improvement on the latent (sub-)constructs and their relations in-between.

Several limitations need to be addressed considering our findings from this study. The implementation of the Mini-RDoC assessment as a core assessment inside the German research network for mental disorders enabled us to build a considerable amount of data providing a transnosological view cutting across known disorder-based categories. However, some diagnostic categories were over- or underrepresented. Thus, while findings are generalizable throughout a large variety of mental diseases future research could validate our findings using a more balanced distribution.

The initial poor model-fit and the need to use variable reduction and modification indices to guide and reshape our a priori assumptions introduced a bias resulting in reduction of robustness and generalizability of our final model. However, we used these methods very carefully and gave detailed information on the changes made. We would like to point out that all changes applied refer to a theoretical basis in our procedures and that model fit was superior to other factor solutions. Nevertheless, future research should replicate our findings to confirm the formed latent structure. Though this procedure may have been a little exploratory, the significantly better fit in comparison to the one factor solution, supports the assumption of a four factor latent structure.

In contrast to the basic assumption of the RDoC approach that latent variables would become apparent across units of analyses (i.e., considering molecules, cells, physiology, circuits, behavior, and self-report) within domains, our approach is mainly symptom-oriented and focuses on the behavioral and self-report units. Therefore, our study did not evaluate cross-unit validity of the RDoC but investigated latent variables within the self-report unit.

Furthermore, our findings on the assignment changes implicated by modification indices and the removal of several constructs due to high error variance should be re-examined and cross-validated in further, preferentially larger, datasets.

Also, there is some ambiguity with respect to the self-report measurements within the RDoC framework [24], suggesting that more research on embedding already validated and reliable self-report measures into the RDoC framework needs to be done, as well as validating new measurements for specific domains that emerged after our initial consensus on the used measurements (e.g., the sensorimotor domain [17]) and their integration with other units of analysis as suggested by f.e. MacNamara and Phan (2016) [55].

To conclude, this study gives a first impression on the latent structure and intercorrelations between four core Research Domain Criteria in a transnosological sample cutting across symptom-based diagnostics. We emphasize the possibility of using already existing and well validated self-report and behavioral measurements to capture aspects of the latent structure formed by the RDoC matrix. This will enable future research connecting the RDoC matrix and its core domains PVS, NVS, CS and SP to outcome measures like disease severity to better characterize domain-specific effects across mental disorders, which may help inform the development of stratified treatment strategies.

## Supplementary Information

Below is the link to the electronic supplementary material.

Supplementary file1 (PDF 161 KB)

Supplementary file2 (PDF 259 KB)

Supplementary file3 (PDF 159 KB)

Supplementary file4 (PDF 165 KB)

Supplementary file5 (PDF 167 KB)

## Data Availability

All original data are on record and accessible to inspection, but are not available publicly based on local and national data protection regulations.

## Code availability

Available from the authors upon request.
