# Supplementary material for: Mapping Research Domain Criteria using a transdiagnostic mini-RDoC assessment in mental disorders: a confirmatory factor analysis
Source: Eur Arch Psychiatry Clin Neurosci. 2022 Jul 1;273(3):527–39. doi: 10.1007/s00406-022-01440-6 (PMC10085934; doi:10.1007/s00406-022-01440-6)
Supplement: Supplementary file 1 — Supplementary file1 (PDF 161 KB) [file 406_2022_1440_MOESM1_ESM.pdf]

Figure SI1

Shell model of assembled Mini-RDoC assessment

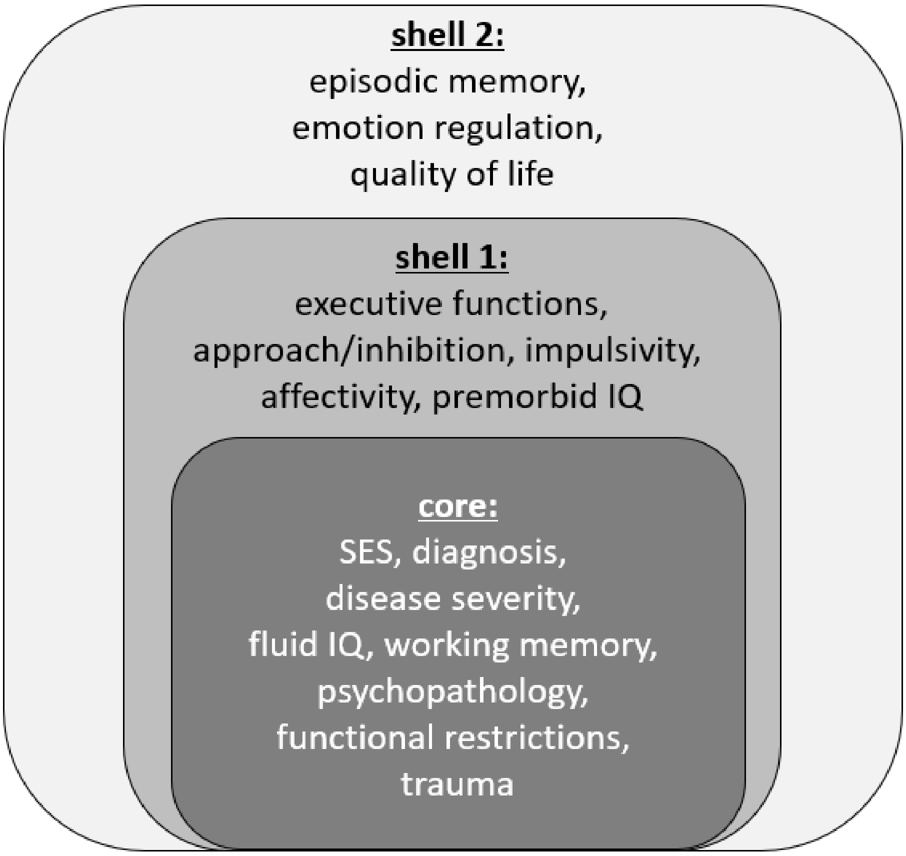

Note. Core had to be implemented inside the assessment process of the study; shells were optional depending on their fit to the specific assessment process of the respective study.

Article: Mapping Research domain criteria using a transdiagnostic Mini-RDoC assessment in mental disorders – a confirmatory factor analysis
Journal: European Archives of Psychiatry and Clinical Neuroscience
Authors: Bernd R. Förstner, Mira Tschorn, Nicolas Reinoso-Schiller, Lea Mascarell Maričić, Erik Röcher, Janos L. Kalman, Sanna Stroth, Annalina V. Mayer, Kristina Schwarz, Anna Kaiser, Andrea Pfennig, André Manook, Marcus Ising, Ingmar Heinig, Andre Pittig, Andreas

Heinz, Klaus Mathiak, Thomas G. Schulze, Frank Schneider, Inge Kamp-Becker, Andreas Meyer-Lindenberg, Frank Padberg, Tobias Banaschewski, Michael Bauer, Rainer Rupprecht, Hans-Ulrich Wittchen, Michael A. Rapp.
Corresponding author: Prof. Dr. med. Dr. phil. Michael A. Rapp, Social and Preventive Medicine, University of Potsdam, Am Neuen Palais 10, 14469 Potsdam, Germany, Phone +49 331 977 4095, Fax +49 331 977 4078, michael.rapp@uni-potsdam.de, Orchid-ID: 0000-0003-0106-966X
