# Supplementary material for: Mapping Research Domain Criteria using a transdiagnostic mini-RDoC assessment in mental disorders: a confirmatory factor analysis
Source: Eur Arch Psychiatry Clin Neurosci. 2022 Jul 1;273(3):527–39. doi: 10.1007/s00406-022-01440-6 (PMC10085934; doi:10.1007/s00406-022-01440-6)
Supplement: Supplementary file 2 — Supplementary file2 (PDF 259 KB) [file 406_2022_1440_MOESM2_ESM.pdf]

Table SI2

Data contributions and Characteristics of the German Research Network of Mental Disorders

Consortium, coordination[a]	Partici-pants	Main aim	Inclusion criteria	Exclusion criteria
**PROTECT-AD**,<br>H.-U. Wittchen,<br>TU Dresden	*N*=600; data used: *N*=600	Optimized extinction: intensified cognitive-behavioral therapy of AD with and without comorbidity	- 15-70 years; ♂, ♀<br>- current primary AD (DSM-V)<br>- severity at baseline HAM-A>18 and CGI>3<br>- able to attend on his/her own or accompanied by significant other<br>- language competence	- general medical contraindications<br>- acute suicidality<br>- any DSM-IV/V psychotic/primary mood disorders (BD I, recurrent or chronic MDD)<br>- mono-symptomatic specific phobia<br>- current substance use dependence<br>- concomitant psychological/psychiatric treatment
**ESCALIFE**,<br>ESCAlate,<br>T. Banaschewski,<br>Central Institute of Mental Health (CIMH) Mannheim	*N*=25; data used: *N*=25	Evidence-based, gradual care of ADHD over the life span	- 16-45years; ♂, ♀<br>- ADHD diagnosis (DSM-V)	- severe heart disease<br>- epilepsy<br>- IQ<80<br>- psychiatric disorders<br>- medication (psychotropic or ADHD): need of a 4-week wash-out period before participation in the study<br>- insufficient language skills<br>- current alcohol or drug dependence<br>- pregnancy or breast feeding<br>- common comorbidities (e.g., conduct disorder, PD (except ASPD); AD or mild to moderate MDD and SUD in remission did not lead to exclusion
**ASD-NET**,<br>I. Kamp-Becker,<br>Philipps University Marburg	*N*=23; data used: *N*=23	ASD over the life span: more effective care through valid diagnoses and a better understanding of etiology	- 19-40 years; ♂<br>- infantile autism<br>- Asperger's syndrome<br>- atypical autism (ICD-10)<br>- native language german	- BMI≤18 or ≥30<br>- IQ<70<br>- investigational product contraindication (e.g., cardiac arrhythmia)<br>- screening results: HR>120 bpm, SBP>180 mmHg, DPD>100 mmHg<br>- medication intake affecting the central nervous system (e.g., opiate, psychotropic drugs)<br>- past neurological and endocrinological diseases<br>- acute suicidality

				- psychiatric disorders<br>- psychotherapeutic treatment<br>- first or second degree relative with ASD<br>- MRI-contraindications<br>- regular consumption of drugs, alcohol, nicotine<br>- AQ-Interview ≥ 32 points
**BipoLife**,<br>M. Bauer,<br>University Hospital Dresden	*N*=99; data used: *N*=99	Improving the detection and treatment of BD	**Dresden**:<br>- 15-35 years; ♂, ♀<br>Risk group I:<br>- consultation of an early recognition center<br>- presence of at least one risk factors for BD: family history, affective symptomatology/ depressive syndrome, hypomanic/ mood swings, disturbances of circadian rhythm/sleep, other clinical hints<br>Risk group II:<br>- in- or outpatients with a depressive syndrome (in the context of MDD, dysthymic disorder, cyclothymic disorder, minor depressive disorder, recurrent brief depressive disorder, adjustment disorder with depressed mood, depressive disorder not otherwise specified)	**Dresden**:<br>- primary diagnosis: BD, SZ, SZA, AD, OCD or SUD<br>- limited ability to comprehend the study<br>- implied expressed negative declaration of intent to participate in the study by a minor<br>- acute suicidality
			**Tübingen:**<br>- 18-55 years; ♂, ♀<br>- current primary BD-I or BD-II (DSM-V)<br>- current on psychiatric treatment with adequate medication<br>- at least 4 weeks of remission<br>- mood disorder (QIDS-C≤10, YMRS≤12)	**Tübingen:**<br>- IQ<85<br>- schizophrenic or other psychotic disorder (eg., ASPD, SZ, SZA or any psychotic disorder); psychotic features within an affective episode are not an exclusion criteria<br>- acute suicidality<br>- substance abuse

			**Munich**[b]:<br>Project A2:<br>- 18-55 years; ♂, ♀<br>- current BD-I or BD-II (DSM-V)<br>- current on psychiatric treatment<br>- appropriate medication according S3-guideline<br>- stable remission (at least 4 weeks)<br>- regarding to the affective episode: clinically relevant limits of external (clinician) rating: (QIDS-C≤10, YMRS≤12)<br>- at least one affective episode in the last 2 years<br>Project B3:<br>- ≥18 years; ♂, ♀<br>- previous or current lithium treatment for a period > 1 year<br>- overall > 4 episodes	**Munich**[b]:<br>- acute suicidality<br>- schizophrenic or other psychotic disorder (DSM-V); Project A2: psychotic characteristics during affective episodes are no criterion for exclusion<br>- IQ <85<br>Project A2:<br>- lack of willingness of prescribed medication intake (normally long-term medication)<br>- receiving current or previous (< 6 months) ambulant standard psychotherapy<br>- SZA, ASPD, or BPD (DSM-V)<br>- SUD (DSM-V, except nicotine) during the last 6 months (earlier SUD and actual stable remissions for over 6 months are allowed)<br>- SUD (DSM-V) endangering study participation<br>Project B3:<br>- clear organic or substance induced cause of the psychiatric disorder
**OptiMD**, R. Rupprecht, University of Regensburg	*N*=360; data used: *N*=171	New strategies for the optimized treatment of depression	- ≥18 years; ♂, ♀<br>**Regensburg, Munich, Heidelberg**:<br>- inpatients with depressive syndrome (HAM-D-21 ≥ 14): first depressive episode, recurrent MDD, BD (current depressive episode), SZA, mixed AD and MDD<br>- caucasian origin<br>**Berlin**:<br>- current primary MDD (ICD-10) during hospital stay	**Regensburg, Munich, Heidelberg**:<br>- severe neurological or internal accompanying illness (e.g., dementia, morbus cushing)<br>- depressive episode secondary to a somatic/neurological disease or substance abuse/dependence<br>- pregnancy or breast-feeding<br>**Berlin**:<br>- control group: current ICD-10 psychiatric disorder

**GCBS**,<br>F. Padberg,<br>University of Munich	*N*=115; data used: *N*=40	Brain stimulation for mental disorders	**Munich**:<br>- 18-65 years; ♂, ♀<br>- MDD (DSM-V), current depressive episode of less than 5 years duration<br>- HAM-D-21≥15 at screening visit<br>- medications: patient did not respond to at least one antidepressant treatment in the current episode; patient is taking a SSRI of adequate dose and ≥ 4 weeks in the current episode	**Munich**:<br>- any relevant instable medical condition<br>- high degree of therapy resistance: >4 sufficient treatment attempts in the current episode<br>- any other relevant psychiatric axis-I- and/or axis-II-disorder<br>- electroconvulsive therapy treatment in the present episode<br>- history of clinical transcranial direct current stimulation treatment (except single experimental sessions)<br>- treatment with deep brain stimulation or vagus nerve stimulation and/or any other intracranial implants<br>- acute suicidality<br>- investigators, site personnel directly affiliated with this study, and their immediate families<br>- pregnancy
			**Berlin**:<br>- 20-65 years; ♂, ♀<br>- MDD (DSM-V), current depressive episode less than 5 years duration<br>- medications: no psychotropic medication (≥4 weeks) or current stable medication (≥ 4 weeks) with SSRIs	**Berlin**:<br>- medication: more than four failed attempts in current episode; other psychotropic medications<br>- outpatient treatment (behavioral therapy) for the current episode<br>- electroconvulsive therapy for the current episode<br>- MRI-contraindications<br>- stroke (past 2 years), epileptic seizure or diagnosed epilepsy, dementia, Parkinson’s disease, Huntington’s chorea, multiple sclerosis, any other neurological disease, which leads to intracranial pressure, brain lesions or higher risk for epileptic seizures<br>- psychotic tendencies in lifetime<br>- BD, AD, PTSD, ED, PD<br>- Substance abuse or dependence in the past 6 months (except nicotine, caffeine)

**APIC**,<br>F. Schneider,<br>RWTH Aachen,<br>Jülich Aachen Research Alliance (JARA-BRAIN)	*N*=295;<br>data used:<br>*N*=89	Antipsychotic-induced structural and functional brain changes	- 18-65 years; ♂, ♀<br>- SZ (DSM-V)<br>- legally competent and capable of taking part in the study	- severe physical diseases (e.g., epilepsy, cancer)<br>- missing or incomplete medication history<br>- MRI-contraindications<br>- pregnancy or breast-feeding<br>- persons placed in an institution on the orders of public authorities or courts<br>- dependency or employment relationship with the sponsor or investigator<br>- simultaneous participation in another clinical trial
**ESPRIT**,<br>A. Meyer-Lindenberg,<br>Central Institute of Mental Health (CIMH) Mannheim	*N*=395;<br>data used:<br>*N*=384	Improving prevention and recovery in schizophrenia through innovative therapies	- 18-65 years; ♂, ♀<br>- patients: SZ, MDD, BD-I, ASD<br>- control group: no personal or family history of psychiatric disorders	- chronic physical disease<br>- MRI-contraindications<br>- patients: BD-II, SUD, PD<br>- patients with comorbidities were not excluded, if comorbidities 1) evolved as a consequence of, or 2) were markedly less pronounced as the primary disorder

Note. Characteristics of eight consortia of the German Research Network of Mental Disorders. *N* = 1,912 (entry data) and 1,431 (after data preparation, used in analyses). **Consortia**: AERIAL = Early Recognition and Intervention Across the Lifespan; APIC = Antipsychotic induced brain changes; ASD-NET = Autism spectrum disorder - net; ESCAlife = Evidence-based, stepped care of ADHD along the life-span; ESPRIT = Enhancing schizophrenia prevention and recovery through innovative treatments; GCBS = German center for brain stimulation; OptiMD = Optimized treatment of major depression; PROTECT-AD = Providing tools for effective care and treatment of anxiety disorders. **Disorders**: AD = Anxiety disorder; ADHD = Attention deficit hyperactivity disorder; ASD = Autism spectrum disorder; ASPD = Antisocial personality disorder, BD = Bipolar disorder; BPD = Borderline personality disorder; ED = Eating disorder; MDD = Major depression disorder; OCD = Obsessive-compulsive disorder; PD = Personality disorder; PTSD = Posttraumatic stress disorder; SUD = Substance use disorder; SZ = Schizophrenia; SZA = Schizoaffective disorder. **Other**: AQ = Autism-Spectrum Quotient; DPD = Diastolic blood pressure; DSM-V/DSM-IV= Diagnostic and Statistical Manual of Mental Disorders, Fourth/Fifth Edition; CGI = Clinical Global Impression Scale; HAM-A = Hamilton Anxiety Rating Scale; HAM-D-21 = Hamilton Depression Rating Scale, Version 21; HR = Heart rate; ICD-10 = International Classification of Diseases, 10th Revision; MRI = Magnet Resonance Imaging; QIDS-C = Quick Inventory of Depression Symptomatology - Clinician rating; SBP = Systolic blood pressure; SSRI = Selective Serotonin Reuptake Inhibitor; YMRS = Young Mania Rating Scale.

[a] Coordination center for each consortium (for details of all consortia members, see Bauer et al. (20))

[b] Location provided data of two subprojects: Bipolife A2 and Bipolife B3

Article: Mapping Research domain criteria using a transdiagnostic Mini-RDoC assessment in mental disorders – a confirmatory factor analysis
Journal: European Archives of Psychiatry and Clinical Neuroscience
Authors: Bernd R. Förstner, Mira Tschorn, Nicolas Reinoso-Schiller, Lea Mascarell Maričić, Erik Röcher, Janos L. Kalman, Sanna Stroth, Annalina V. Mayer, Kristina Schwarz, Anna Kaiser, Andrea Pfennig, André Manook, Marcus Ising, Ingmar Heinig, Andre Pittig, Andreas Heinz, Klaus Mathiak, Thomas G. Schulze, Frank Schneider, Inge Kamp-Becker, Andreas Meyer-Lindenberg, Frank Padberg, Tobias Banaschewski, Michael Bauer, Rainer Rupprecht, Hans-Ulrich Wittchen, Michael A. Rapp.
Corresponding author: Prof. Dr. med. Dr. phil. Michael A. Rapp, Social and Preventive Medicine, University of Potsdam, Am Neuen Palais 10, 14469 Potsdam, Germany, Phone +49 331 977 4095, Fax +49 331 977 4078, michael.rapp@uni-potsdam.de, Orchid-ID: 0000-0003-0106-966X
