# Supplementary material for: Mapping Research Domain Criteria using a transdiagnostic mini-RDoC assessment in mental disorders: a confirmatory factor analysis
Source: Eur Arch Psychiatry Clin Neurosci. 2022 Jul 1;273(3):527–39. doi: 10.1007/s00406-022-01440-6 (PMC10085934; doi:10.1007/s00406-022-01440-6)
Supplement: Supplementary file 3 — Supplementary file3 (PDF 159 KB) [file 406_2022_1440_MOESM3_ESM.pdf]

Table SI3

Construction of the PD-CAN-Assessment as a shell model

RDoC construct	Units of Analysis	Instrument	Abbreviation
**Core**			
SES, medication	Anamnesis	Standardized interview	
Diagnosis	Anamnesis	Standardized interviews and checklists [a]	
Fluid intelligence	Behavior	Digit Symbol SubstitutionTest	DSST
Working memory	Behavior	Digit span (forward)	DF
Psychopathology	Self-report	Brief Symptom Inventory	BSI-53
Functional restriction	Self-report	WHO Disability Assessment Schedule 2.0	WHO-DAS 2.0
Childhood Trauma	Self-report	Childhood Trauma Screener	CTS
**Shell 1**			
Cognitive speed processing	Behavior	Trail Making Test A	TMT-A
Executive functioning	Behavior	Trail Making Test B	TMT-B
Approach	Self-report	Behavioral Approach Scale	BIS/BAS
Inhibition	Self-report	Behavioral Inhibition Scale	BIS/BAS
Impulsivity	Self-report	Barratt Impulsiveness Scale – Short Version	BIS-15
Affect	Self-report	Positive and Negative Affect Schedule	PANAS
Premorbid Intelligence	Behavior	Multiple-Choice Word Test – Version B	MWT-B
**Shell 2**			
Episodic Memory	Behavior	Verbal Learning and Memory Test	VLMT
Emotion Regulation	Self-report	Emotion Regulation Questionnaire	ERQ
Quality of Life	Self-report	WHO- 5 Well-Being Index	WHO-5

Note. SES = Socioeconomic status; RDoC = Research Domain Criteria
[a] Standardized interviews and checklists: SCID (Structured Clinical Interview for Diagnostic and Statistical Manual of Mental Disorders [DSM-IV]), CIDI (Composite International Diagnostic Interview), IDCL (International Diagnostic Checklists for International Classification of Diseases [ICD-10]), DIPS (Diagnostic Interview for Mental Disorders) or Mini-DIPS.

Article: Mapping Research domain criteria using a transdiagnostic Mini-RDoC assessment in mental disorders – a confirmatory factor analysis
Journal: European Archives of Psychiatry and Clinical Neuroscience
Authors: Bernd R. Förstner, Mira Tschorn, Nicolas Reinoso-Schiller, Lea Mascarell Maričić, Erik Röcher, Janos L. Kalman, Sanna Stroth, Annalina V. Mayer, Kristina Schwarz, Anna Kaiser, Andrea Pfennig, André Manook, Marcus Ising, Ingmar Heinig, Andre Pittig, Andreas Heinz, Klaus Mathiak, Thomas G. Schulze, Frank Schneider, Inge Kamp-Becker, Andreas Meyer-Lindenberg, Frank Padberg, Tobias Banaschewski, Michael Bauer, Rainer Rupprecht, Hans-Ulrich Wittchen, Michael A. Rapp.
Corresponding author: Prof. Dr. med. Dr. phil. Michael A. Rapp, Social and Preventive Medicine, University of Potsdam, Am Neuen Palais 10, 14469 Potsdam, Germany, Phone +49 331 977 4095, Fax +49 331 977 4078, michael.rapp@uni-potsdam.de, Orchid-ID: 0000-0003-0106-966X
