# Supplementary material for: Mapping Research Domain Criteria using a transdiagnostic mini-RDoC assessment in mental disorders: a confirmatory factor analysis
Source: Eur Arch Psychiatry Clin Neurosci. 2022 Jul 1;273(3):527–39. doi: 10.1007/s00406-022-01440-6 (PMC10085934; doi:10.1007/s00406-022-01440-6)
Supplement: Supplementary file 4 — Supplementary file4 (PDF 165 KB) [file 406_2022_1440_MOESM4_ESM.pdf]

Table SI4

Additional changes on scales and items with descriptive statistics

RDoC	Old variable	Items	Transformation	New variable	*Mean*	*SD*
PVS	BIS/BAS subscales Drive and Reward Responsiveness	3, 7, 12, 21	Forming mean score	Scale Goal Attainment	2.90	0.58
PVS	PANAS subscale Positive Affect	active, interested, enthusiastic, determined	Item reduction and forming mean score	Scale Hedonic Affect	1.78	0.82
PVS	BSI-53 single items Obsessive-compulsive	15r, 26r, 27r	Forming mean score	Scale Habituation	2.99	0.86
NVS	BIS/BAS subscale Behavioral Inhibition	2r, 8, 22r, 24	Forming mean score	Scale Anxiety-based Inhibition	3.23	0.59

Note. Variables were transformed with log10 to address normal distribution issue. *SD* = Standard deviation; r = reversed; RDoC = Research Domain Criteria; PVS = Positive valence systems; NVS = Negative valence systems; BIS/BAS = Behavioral Inhibition System/Behavioral Activation System Scale; BSI-53 = Brief Symptom Checklist; PANAS = Positive and Negative Affect Scale.

Article: Mapping Research domain criteria using a transdiagnostic Mini-RDoC assessment in mental disorders – a confirmatory factor analysis
Journal: European Archives of Psychiatry and Clinical Neuroscience
Authors: Bernd R. Förstner, Mira Tschorn, Nicolas Reinoso-Schiller, Lea Mascarell Maričić, Erik Röcher, Janos L. Kalman, Sanna Stroth, Annalina V. Mayer, Kristina Schwarz, Anna Kaiser, Andrea Pfennig, André Manook, Marcus Ising, Ingmar Heinig, Andre Pittig, Andreas Heinz, Klaus Mathiak, Thomas G. Schulze, Frank Schneider, Inge Kamp-Becker, Andreas Meyer-Lindenberg, Frank Padberg, Tobias Banaschewski, Michael Bauer, Rainer Rupprecht, Hans-Ulrich Wittchen, Michael A. Rapp.
Corresponding author: Prof. Dr. med. Dr. phil. Michael A. Rapp, Social and Preventive Medicine, University of Potsdam, Am Neuen Palais 10, 14469 Potsdam, Germany, Phone +49 331 977 4095, Fax +49 331 977 4078, michael.rapp@uni-potsdam.de, Orchid-ID: 0000-0003-0106-966X
