# Supplementary material for: Mapping Research Domain Criteria using a transdiagnostic mini-RDoC assessment in mental disorders: a confirmatory factor analysis
Source: Eur Arch Psychiatry Clin Neurosci. 2022 Jul 1;273(3):527–39. doi: 10.1007/s00406-022-01440-6 (PMC10085934; doi:10.1007/s00406-022-01440-6)
Supplement: Supplementary file 5 — Supplementary file5 (PDF 167 KB) [file 406_2022_1440_MOESM5_ESM.pdf]

Table SI5

Model comparison: Fit indices and X^2-test

Model no.	Model characteristics	CFI	TLI	RMSEA (90% CI)	Comparison	X^2 (df diff), (p)
1	4 factor model with a priori variables (transformed if necessary)	.77	.75	.078 (.076 - .081)		
2	Model 1 with 1 factor only	.69	.67	.090 (.088 - .093)	1 vs. 2	(6) 1158.1 (<.001)
3	Model 1: no covariances allowed between the factors	.59	.56	.104 (.102 - .106)	1 vs. 3	(6) 2571.2 (<.001)
4	4 factor model with reduced/changed variables (transformed if necessary)	.93	.92	.077 (.072 - .082)		
5	Model 4 with 1 factor only	.78	.74	.136 (.131 - .140)	4 vs. 5	(6) 1656.3 (<.001)
6	Model 4 with no covariances allowed between the factors	.71	.66	.154 (.149 - .158)	4 vs. 6	(6) 2327.8 (<.001)

Note. CFI = Comparative Fit Index; CI = Confidence Interval; df diff = degrees of freedom difference; TLI = Tucker Lewis Index; RMSEA = Root Mean Square Error of Approximation.

Article: Mapping Research domain criteria using a transdiagnostic Mini-RDoC assessment in mental disorders – a confirmatory factor analysis
Journal: European Archives of Psychiatry and Clinical Neuroscience
Authors: Bernd R. Förstner, Mira Tschorn, Nicolas Reinoso-Schiller, Lea Mascarell Maričić, Erik Röcher, Janos L. Kalman, Sanna Stroth, Annalina V. Mayer, Kristina Schwarz, Anna Kaiser, Andrea Pfennig, André Manook, Marcus Ising, Ingmar Heinig, Andre Pittig, Andreas Heinz, Klaus Mathiak, Thomas G. Schulze, Frank Schneider, Inge Kamp-Becker, Andreas Meyer-Lindenberg, Frank Padberg, Tobias Banaschewski, Michael Bauer, Rainer Rupprecht, Hans-Ulrich Wittchen, Michael A. Rapp.
Corresponding author: Prof. Dr. med. Dr. phil. Michael A. Rapp, Social and Preventive Medicine, University of Potsdam, Am Neuen Palais 10, 14469 Potsdam, Germany, Phone +49 331 977 4095, Fax +49 331 977 4078, michael.rapp@uni-potsdam.de, Orchid-ID: 0000-0003-0106-966X
